# A vulnerability index for COVID-19: spatial analysis at the subnational level in Kenya

**DOI:** 10.1136/bmjgh-2020-003014

**Published:** 2020-08-23

**Authors:** Peter M Macharia, Noel K Joseph, Emelda A Okiro

**Affiliations:** 1 Population Health Unit, KEMRI-Wellcome Trust Research Programme, Nairobi, Kenya; 2 Centre for Tropical Medicine and Global Health, Nuffield Department of Clinical Medicine, University of Oxford, Oxford, UK

**Keywords:** public health, indices of health and disease and standardisation of rates, geographic information systems, epidemiology

## Abstract

**Background:**

Response to the coronavirus disease 2019 (COVID-19) pandemic calls for precision public health reflecting our improved understanding of who is the most vulnerable and their geographical location. We created three vulnerability indices to identify areas and people who require greater support while elucidating health inequities to inform emergency response in Kenya.

**Methods:**

Geospatial indicators were assembled to create three vulnerability indices; Social VulnerabilityIndex (SVI), Epidemiological Vulnerability Index (EVI) and a composite of the two, that is, Social Epidemiological Vulnerability Index (SEVI) resolved at 295 subcounties in Kenya. SVI included 19 indicators that affect the spread of disease; socioeconomic deprivation, access to services and population dynamics, whereas EVI comprised 5 indicators describing comorbidities associated with COVID-19 severe disease progression. The indicators were scaled to a common measurement scale, spatially overlaid via arithmetic mean and equally weighted. The indices were classified into seven classes, 1–2 denoted low vulnerability and 6–7, high vulnerability. The population within vulnerabilities classes was quantified.

**Results:**

The spatial variation of each index was heterogeneous across Kenya. Forty-nine northwestern and partly eastern subcounties (6.9 million people) were highly vulnerable, whereas 58 subcounties (9.7 million people) in western and central Kenya were the least vulnerable for SVI. For EVI, 48 subcounties (7.2 million people) in central and the adjacent areas and 81 subcounties (13.2 million people) in northern Kenya were the most and least vulnerable, respectively. Overall (SEVI), 46 subcounties (7.0 million people) around central and southeastern were more vulnerable, whereas 81 subcounties (14.4 million people) were least vulnerable.

**Conclusion:**

The vulnerability indices created are tools relevant to the county, national government and stakeholders for prioritisation and improved planning. The heterogeneous nature of the vulnerability indices underpins the need for targeted and prioritised actions based on the needs across the subcounties.

**Key questions**

**What is already known?**

- Disasters and adverse health events such as epidemics and pandemics disproportionately affect population with significantly higher impacts on the most vulnerable and less resilient communities.
- Significant health, socioeconomic, demographic and epidemiological disparities exist within Kenya when considering individual determinants, however, little is known about the spatial variation and inequities of their concurrence.

**What are the new findings?**

- Subcounties in northwestern and partly eastern Kenya are most vulnerable when considering the social vulnerability index, whereas central and southeast regions are most vulnerable based on the epidemiological vulnerability index affecting approximately 6.9 million and 7.2 million people, respectively.
- The combined index of social and epidemiological vulnerabilities (SEVI) shows that on average, 15% (7.0 million) of Kenyans reside in the most vulnerable subcounties mainly located in the central and southeastern parts of Kenya.

**What do the new findings imply?**

- Targeted and prioritised actions that cushion against negative effects on the most vulnerable population are essential to respond to the current COVID-19 pandemic.
- Need for strategies that address the socioeconomic determinants of health due to high levels of socioeconomic deprivation. The indices are valuable tools for use within the health system to identify areas that need strengthening such as shortage of health workers and inadequate health infrastructure against the projected population in need.
- Need for better quality data to define a robust vulnerability index at a high spatial resolution that can be adapted and used in response to future disasters and adverse health events in the long run.

## Introduction

There has been growing recognition of the threat that epidemics, disasters and public health emergencies pose to global health security and the livelihoods of people, beyond their impact on human health.^1^ Under the umbrella of the global health security agenda, countries have come together to advance a world safe and secure from infectious disease threats. Hence, countries are expected to be prepared to weather disease outbreaks and natural disasters with Africa having a history of dealing with emerging diseases^2 3^ most recently the Ebola epidemic in West Africa and Democratic Republic of the Congo.^4^

Epidemics disproportionally impact vulnerable populations as can be witnessed with the coronavirus disease 2019 (COVID-19) in the USA and the UK where black, Asian and minority ethnic communities are being disproportionately affected^5–7^ as are poorer communities^8 9^; during the Ebola outbreak, the disease was linked to rural and remote areas.^10^ The reasons behind this disparity are complex and varied; however, one of the biggest underlying factors driving this disproportionate impact is socioeconomic deprivation.^8 11^ Several factors, including poverty, lack of access to healthcare and transportation, and certain aspects of housing may weaken a community’s ability to prevent significant human and financial loss when a disaster such as the current COVID-19 pandemic strikes. A framework that provides a detailed understanding and location of population groups that are either vulnerable to increased risk of infection or inferior health outcomes and that are also marginalised from health services can inform where additional resources are most needed. These factors are collectively identified as vulnerability and aim to identify people who are disproportionally exposed to the risk of infection and/or disease severity.

COVID-19 rapidly spread worldwide and although low testing numbers do not allow for a reliable appraisal of the extent of the epidemic in Africa, 115 616 cases and over 3479 deaths had been reported in Africa as of 26 May 2020.^12^ Health officials expect that it is only a matter of time before infections begin to rise in Africa with growing concerns about risks in a continent that faces unique challenges in dealing with the current outbreak.^2^ WHO has estimated that between 83 000 and 190 000 people could die of COVID-19 and 29–44 million could get infected during the first year of the pandemic if containment measures fail in Africa.^13^ However, although cases are increasing, most countries in Africa are not documenting the same exponential growth rate in confirmed cases as seen in Europe and the USA.^11 14^

The first case of COVID-19 in Kenya was confirmed on 13 March 2020. Since then, 1348 cases have been reported with 52 deaths confirmed by 26 May 2020.^15^ The Kenyan government, through the Ministry of Health, has responded quickly by taking proactive public health measures to combat the spread of the disease. These measures have been through intensive tracking of contacts, isolation of confirmed cases, halting non-essential services and cutting off routes of transmission through suspension of international flights, dusk-to-dawn curfew, partial lockdowns and most recently closing borders with high-intensity neighbouring countries of Tanzania and Somalia.^15^ However, certain challenges face the implementation of some of these measures. There has been widespread growth of informal settlements in Kenya that are typically characterised by high-density neighbourhoods and poor infrastructure common across the continent where self-isolation and social distancing are proving extremely challenging. A substantial number of people work in the informal sector^16^ where working from home is not realistic and the risks of a sudden loss of income are a constant threat with many more suffering from insecure food supplies.^17^

People living in these settings often have malnutrition, infectious diseases such as HIV/AIDS and some may suffer from non-communicable diseases (NCDs) putting these individuals at greater risk of more severe clinical infection and mortality due to COVID-19.^18^ Other challenges include high poverty rates, weaker health systems and minimal access to healthcare with most countries having inadequate financial leeway to provide an effective response without external assistance. Although the scarcity of resources is a matter of concern, it is equally important to look at whether the resources available support the most affected communities. This time presents an opportunity to reduce overall inequities by supporting the most vulnerable groups. Some level of prioritisation and targeting of resources will prove useful in this context to identify those people and places that face the highest risk.

Using a wide range of spatially referenced indicators to enumerate a varied range of social constraints and assess risk, we developed three COVID-19-specific vulnerability indices to enumerate social vulnerability (affects the risk of infection and spread), epidemiological vulnerability (affects the risk of progression to severe disease) and a combination of the two indices defined at the subcounty level in Kenya. This will facilitate the identification of vulnerable groups and elucidate health inequities to help public health response by identifying and mapping communities that will most likely need greater support during the current outbreak.

## Methods

### Country context

The Republic of Kenya located in Eastern Africa covers approximately 591 971 km^2^ and lies on the equator across the great East African Rift Valley, extending from Lake Victoria to Lake Turkana and further southeast to the Indian Ocean (online supplementary additional file 1). Kenya has 47.6 million people with an intercensal growth rate of 2.2% between 2009 and 2019.^16^ Human settlement within Kenya is overdispersed. The highest densities are around the Lake Victoria, the western and central highlands, Nairobi corridor and the main coastal areas, whereas southern and northern areas are sparsely populated.^16^ Kenya has over 300 urban areas with Nairobi and Mombasa counties considered entirely urban.^16^ The average household size in Kenya was 3.9 persons in 2019.^16^

10.1136/bmjgh-2020-003014.supp1 Supplementary data

Approximately 30% of Kenyan live in informal settlements (slums, squatter settlements or shanties).^19^ The Kenyan informal sector employed approximately 15.1 million people in 2019 covering mainly small-scale normally semiorganised, unregulated activities and use low and simple technologies.^20^ According to the latest census data (2019), 7.1 million people have never been to school and 2.6 million are seeking work (employment). Only a third (34.2%) of the households have access to piped water and 8.2% do not have access to any sanitation facility. More than half (55.1%) of the households use firewood as the main type of cooking fuel and only 7.7% own a motor vehicle.^16^

In Kenya, NCDs account for approximately 27% of the total deaths and over 50% of total hospital admissions. The major NCDs in Kenya are cardiovascular conditions, cancer, diabetes and chronic obstructive pulmonary diseases with their sequelae.^21^ The prevalence of health conditions and comorbidities is variable. National adult HIV prevalence was 4.9% in 2017^22^ and diabetes and hypertension prevalence was 2.4% and 23.8%, respectively, in 2015.^21 23^ About 13% of Kenyans consumed some form of tobacco product, while 27% were either overweight or obese in 2015.^21^

### Geographic analysis unit

In 2013, Kenya adopted a decentralised system of governance where the units of administration and health planning were revised to 47 counties with broad policy directions maintained at the national level.^24 25^ The counties are further subdivided into subcounties^26^ and were adopted as the unit of analysis. A geospatial layer of all subcounties was created by digitising subcounty maps available for each county from the county integrated development plans (CIDPs).^26^ The online supplementary additional file 1 shows all the 295 subcounties of Kenya derived from the CIDPs.

### Data assembly

Three indices were defined, Social Vulnerability Index (SVI), Epidemiological Vulnerability Index (EVI) and a composite of the two, the Social Epidemiological Vulnerability Index (SEVI). SVI broadly refers to the resilience of communities in the face of external stresses such as disasters and disease outbreaks on human health.^27^ The indicators used to define SVI entail socioeconomic deprivation,^17 28 29^ population dynamics^28^ and access to services^28–31^ that are likely to affect the risk of infection and spread of the disease at different rates in different subnational areas.

The EVI encapsulates diseases and comorbidities that affect the likelihood of disease progression hence affecting the severity of COVID-19 disease.^18 32–35^ They include underlying medical conditions such as liver disease, chronic kidney disease, obesity, hypertension, diabetes, smoking, cardiovascular disease, chronic respiratory disease and cancer and people who are immunocompromised including HIV/AIDS.^18 32–35^ The SVI and EVI were combined to generate SEVI to explore the overall resilience and risk of developing severe COVID-19 disease in Kenya.

To create the indices at the subnational level in Kenya, 24 data layers were assembled from various sources (table 1). The choice of the data was based on preliminary findings suggesting an association of an indicator with COVID-19 disease,^17 18 28–35^ the availability of the corresponding data layer at the spatial resolution of analysis and localising to Kenya’s socioeconomical and epidemiological context. Nineteen datasets were used to define the SVI, whereas 5 were used to construct the EVI. The datasets were available in different formats and at different spatial resolution, necessitating preprocessing and/or modelling before being input to the framework for constructing a vulnerability index (table 1).

**Table 1:** Data layers used to define COVID-19 vulnerability index in Kenya including their definition, sources, spatial resolution, maximum and minimum values, year of they refer to and preprocessing done (the layers are classified into four thematic areas)

Index	Subindex	Domain	ID	Indicator and definition	Definition	Maximum–minimum	Year/source	Resolution
SEVI	SVI	Socioeconomic deprivation	1	Informal employment​	Percent of adults (aged 15–49 years) who work in a manual labour profession such as construction worker and motor vehicle driver	0.00–15.11	2014^36^	1×1 km
			2	Detergent availability	Percent of households where no soap/detergent was observed	0–97.46		
			3	Car ownership	Percent living in a household that does not own a private car​	53.90–99.96		
			4	Place for handwashing	Percent living in a household with no place for handwashing	4.92–95.14		
			5	Education attainment​	Mean years of school/education attainment​	2.95–21.73	2015^37^	5×5 km
			6	Unimproved water source​	Proportion of households without access to improved water sources​	0.73–95.91	2014^38 39^	
			7	Malnutrition​	Prevalence of stunting among children	21.87–51.34	2015^40^	
			8	Poor households	Proportion of households within the poorest and poorer wealth quintile​	0.44–97.62	DHS 2014 47	Subcounty(B)
			9	Shared sanitation facilities​	Percentage in the households sharing a toilet facility​	4.07–95.67		
		Population characteristics	10	Informal settlements	Percentage of people living in informal settlements and IDP camps	0.00–81.52	2019^86–88^	See footnote
			11	Elderly population​	Percentage of the population aged 65+​ years	0.61–6.30	2019^41 42^	1×1 km
			12	Single-parent families​	Percentage of the population headed by a single parent​	4.56–35.32	DHS 2014 47	Subcounty (B)
			13	Crowded households​	Percentage in the population with 3+ persons per bedroom​	17.06–88.97		
			14	Log Population density	Log of the total population per unit area	−0.24 to 5.10	2019^16^	See footnote
			15	Urban population	Proportion of population living in urban areas	0.00–100.00		
		Access to services	16	Access to hospitals	Proportion of population outside 2 hours travel of a hospital	0.00–100.00	2019^43^	1×1 km
			17	Health workforce	Number of clinicians and medical officers per population​	0.00–152.2	2019^16 89^	See footnote
			18	Hospital beds​	Number of hospital beds per population​	0.00–152.06		
			19	Access to urban areas	Travel time to the nearest urban centre with at ≥5000 people	0.00–3641	2015^44^	1×1 km
	EVI	Epidemiological factors	20	HIV	HIV prevalence among adults​	0.62–22.67	2017^45^	5×5 km
			21	Smoking​	Percent of households with least a daily or weekly smoker	0.70–28.92	2014^36^	1×1 km
			22	Obesity​	​Percentage of adults categorised as obese	0.00–38.62	NCD survey 2015 48	County (B)
			23	Diabetes​	Percentage of adults diagnosed with diabetes​	0.00–17.71		
			24	Hypertension​	Percentage of adults diagnosed with high blood pressure ​	0.00–53.70		

The methods used to create each dataset are detailed in the references provided in table 1 and fall into three broad categories. (1) Methods used to create gridded surfaces (denoted with a spatial resolution of either 1×1 km or 5×5 km), using model-based geostatistics that produces probabilistic inference on a spatially continuous phenomenon based on data collected over a finite set of geo-referenced locations^90^ or the use of a cost distance algorithms to define travel time (spatial access) to either the nearest urban area or health facility.^43 44^ (2) Generated from household surveys using small area estimation (SAE) techniques (denoted by letter B). SAE smooths estimates by borrowing strength from adjacent units and weights predictions towards the estimated prevalence of neighbouring areas. The spatial dependence was represented through a queen adjacency^91^ and modelled via the Besag-York-Molliè 2 conditional autoregressive model. (3) The remaining datasets required a combination of steps to derive them. Lists of informal settlements were extracted from online portals^86 87^ and their extents digitised using Google Earth. Population density maps^88^ were used to define the proportion of the population living in the digitised settlements by subcounty. Similarly, the proportion of the population living in urban areas by subcounty was derived based on a list of urban areas according to Kenya’s 2019 census.^16^ Population per unit area (population density) were available by subcounty,^16^ whereas the number of health workers and hospital beds per population available from the 2018 Kenya harmonised health facility assessment^89^ were divided by the total population per subcounty^16^ to derive health workers and beds per 10 000 population. We considered hospitals bed instead of ICU beds that are very few in Kenya.^31^ When hospital beds are augmented with oxygen supply, they offer an essential requirement for the management of patients with COVID-19 in resource-constrained countries such as Kenya.^31^

EVI, Epidemiological Vulnerability Index ; ICU, intensive care unit; IDP, internally displaced people; NCD, non-communicable disease; SEVI, Social Epidemiological Vulnerability Index ; SVI, Social Vulnerability Index.

The 24 layers of data fall into three broad groups. The first group comprised of 12 data layers available as gridded surfaces at 1×1 km or 5×5 km spatial resolutions^36–45^ (table 1). The mean value per indicator and subcounty was extracted using the *zonal statistics* function of the Spatial Analyst tool of ArcMap V.10.5 (ESRI, Redlands, CA, USA). The second group comprised seven variables whose coverage was estimated using small area estimation (SAE) models^46^ based on the 2014 demographic and health survey (DHS)^47^ and the 2015 stepwise survey for NCDs^48^ conducted in Kenya (table 1, denoted as B). DHS 2014 and NCDs survey were designed to provide precise estimates at the county and national levels, respectively. Therefore, SAE models were used to smooth estimates at subcounty (DHS 2014) and county level (NCDs survey) using R-INLA package^49^ in R software (V.3·4·1). The remaining five data layers were derived through a combination of geospatial techniques (table 1 footnote).

### Constructing SVI

COVID-19 disease vulnerability indices have been created using different approaches ranging from technical^50–52^ to simpler approaches.^53–55^ In this analysis, the indices were created following approaches used to define universal health coverage (UHC) and equity in maternal and child health,^56–60^ infectious disease vulnerability index^61^ and some of the recent COVID-19 vulnerability indices.^53–55^

**Figure 1:**
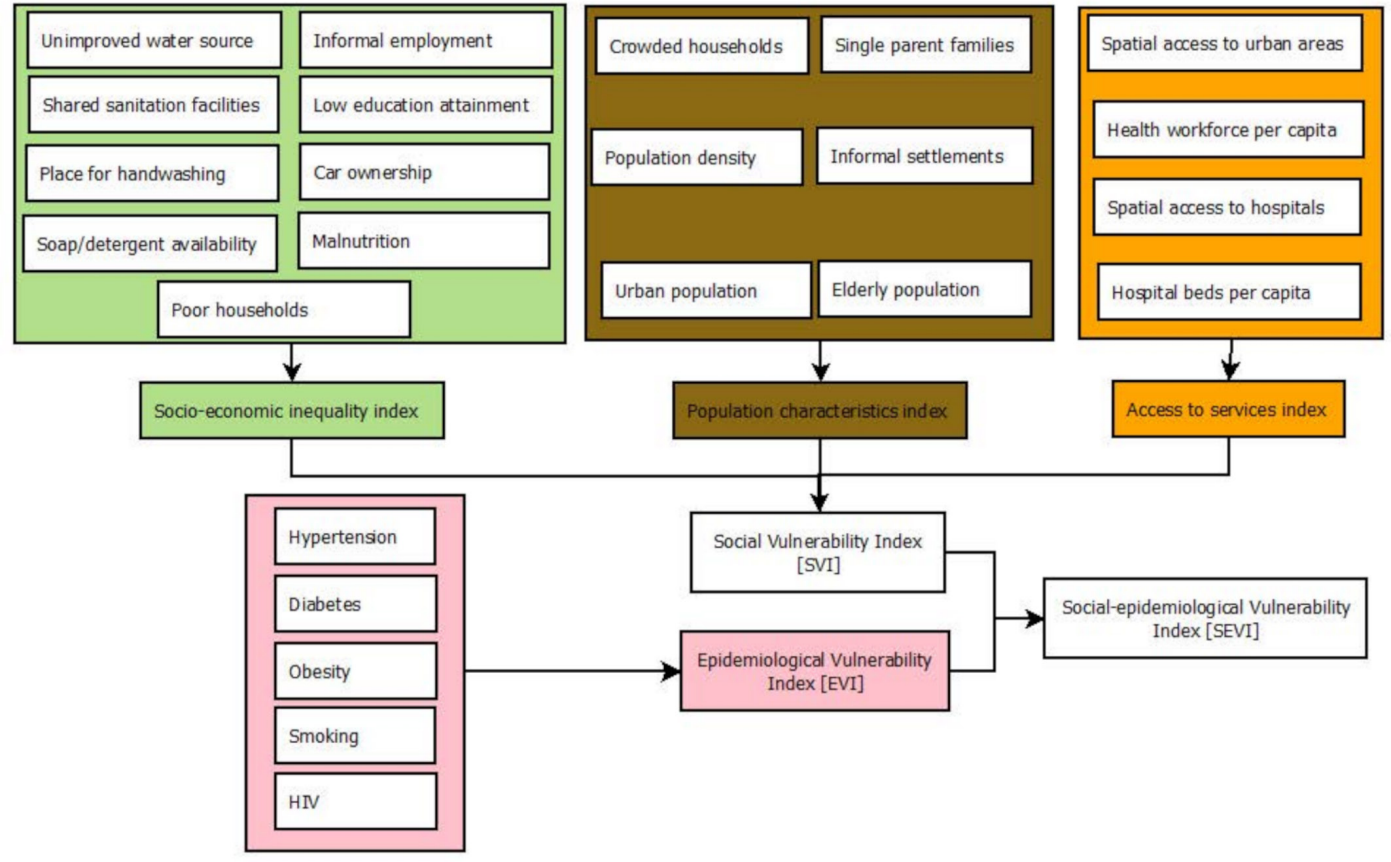
Schematic representation of data layers and approaches used to define Social Vulnerability Index (SVI), Epidemiological Vulnerability Index (EVI) and the combination of the two, Social Epidemiological Vulnerability Index (SEVI) at the subnational level in Kenya.

To create SVI for Kenyan subcounties, three subdomain indices were first defined based on major thematic areas related to COVID-19 vulnerability (table 1 and figure 1). They included socioeconomic deprivation, population characteristics and access to services. The subdomains facilitate a finer and detailed viewpoint that would have been masked by a single averaged index. A similar approach was used when creating an infectious disease vulnerability index for African countries^61^ and a composite coverage index for measuring UHC.^58^

Nine indicators (table 1 and figure 1) were used to define the socioeconomic deprivation subdomain. People of low socioeconomic status including those under casual employment and physical labour are unlikely to have resources to actualise measures put in place by the government such as social distancing viz-a-viz earning a daily livelihood.^7 17 29^ Furthermore, households need to have access to a basic handwashing facility, soap and detergents as a first line of protection against infection.^17^ The population characteristics subdomain included six indicators related to population dynamics (table 1 and figure 1). As population density increases, the rate of transmission of infectious diseases increases,^62^ and informal settlements, refugee and internally displaced people (IDP) camps are vulnerable mainly due to shared community facilities such as water points and toilets, whereas rural areas maybe at a lower risk.^63^ The risk of COVID-19 increases with age especially after 60 years.^17 18 64^

Finally, four indicators were used to define access to services (table 1, figure 1). About 8% of the Kenyan population lives outside 2-hour travel of the nearest facility capable of offering hospital and emergency care^43^; that might be needed to deal with severe COVID-19 cases with significant gaps in hospital capacity to accommodate a surge due to COVID-19.^31^ Further, commodities and services that might be needed by people during the COVID-19 era are likely to be concentrated in urban areas with marginalised areas associated with poorer health and education outcomes.^44^

The geospatial data layers in each subdomain had different scales with different minima and maxima values (table 1). Therefore, to make the values comparable, they were first rescaled to a common scale ranging between 0 (least vulnerable) and 100 (most vulnerable) (equation 1). The scaled indicators were used to create a unique index for each of the three subdomains through the arithmetic mean and were equally weighted. The arithmetic mean of the three subdomains indices was used to create the SVI (equation 2).

#### Equation 1

Rescaling of coverage and or prevalence values for each determinant to a common scale ranging from 0 (least vulnerable) to 100 (most vulnerable)

$$Scaled\;value=\left(\frac{m-r_{min}}{r_{max}-r_{min}}\right)*\left(t_{max}-t_{min}\right)+t_{min}$$

where m is the value to be scaled; r_min_ is the minimum value in the original range (table 1); r_max_ is the maximum value in the original range (table 1); t_min_ is the minimum value in the new scale (0) and t_max_ is the maximum value in the new scale (100).

The choice of weighting scheme for an analysis shapes the outcome (index) with several schemes to consider. The variables can be weighted equally, using pre-existing weights based on literature review, deriving weights from expert opinion (policy-makers, researchers and citizens) or computed using a statistical approach such as the amount of variation a predictor explains in the outcome.^56 58 65 66^ However, there is no gold-standard recommended approach. An equal weighting scheme was implemented for all the determinants in this analysis because the data were insufficient and at a coarse spatial resolution to allow the derivation of weights using statistical approaches. The body of knowledge on the risk factors of COVID-19 and how they might impact COVID-19 is at infancy and not well developed because the pandemic is still unravelling. Equal weighting schemes have been used successfully in creating indices related to infectious disease,^61^ UHC^67 68^ maternal and child health.^69 70^ It has also been shown that the index created through principal component analysis is highly correlated with an index created through various weighting schemes and that arbitrarily defined weights did not make any difference to the composite coverage index.^60 71^ In Mexico, a comparison of equal weights and weighting based on the total health gains yielded similar results.^65^

#### Equation 2

Computation of SVI by combining three subdomains encompassing socioeconomic deprivation (nine indicators), population characteristics (six indicators) and access to services (four indicators) through equal weighting. All the 19 indicators were first scaled (equation 1).

$$SVI=\left[\frac{\left(\frac{Social-economic\;indictors}{9}\right)+\left(\frac{Population\;indicators}{6}\right)+\left(\frac{Access\;to\;services\;indictors}{4}\right)}{3}\right]$$

### Constructing EVI and SEVI

Five indicators on underlying health conditions and comorbidities, risk factors for the severe disease^18 32–35^ were available including the prevalence of HIV, smoking, obesity, diabetes and hypertension (table 1, figure 1). Similarly, these layers were first rescaled (equation 1) and spatially overlaid using the arithmetic mean and equally weighted to generate EVI (equation 3). Finally, the generated SVI and EVI were averaged to generate the composite final index, SEVI (equation 4).

#### Equation 3

Computation of EVI by combining five indicators on underlying health conditions and comorbidities through equal weighting. All the five indicators were first scaled (equation 1).

$$EVI=\left(\frac{HIV+smoking+obesity+diabetes+hypertension}{5}\right)$$

#### Equation 4

Computation of SEVI (SEVI) by combining SVI (equation 1) and EVI (equation 1) through equal weighting.

$$SEVI=\left(\frac{SVI+EVI}{2}\right)$$

Each index was grouped into seven classes ranging from the least vulnerable to the most vulnerable. The classes were grouped using the natural Jenks classification method that identifies ‘natural’ groups within the data by reducing the variance within classes and maximising the variance between classes.^72^ A higher group (the sixth and seventh class) indicated high vulnerability, whereas a lower group (the first and the second class) represented low vulnerability. The groups are unique within each index as the distribution of data among the indices are different. The proportion of the population within each band of vulnerability was then computed based on the 2019 Kenya’s census data.

Figure 1 summarises the input data and methods used to define the indices in Kenya across the 295 subcounties. The analysis and visualisations were done using StataCorp 2014 (Stata Statistical Software: Release V.14; StataCorp LP), R software (V.3·4·1) and ArcMap V.10·5 (ESRI, Redlands, CA, USA).

### Patient and public involvement

Our study does not involve the participation of patients or any members of the public. All data used in this study are aggregated and publicly available as listed in table 1.

## Results

**Figure 2:**
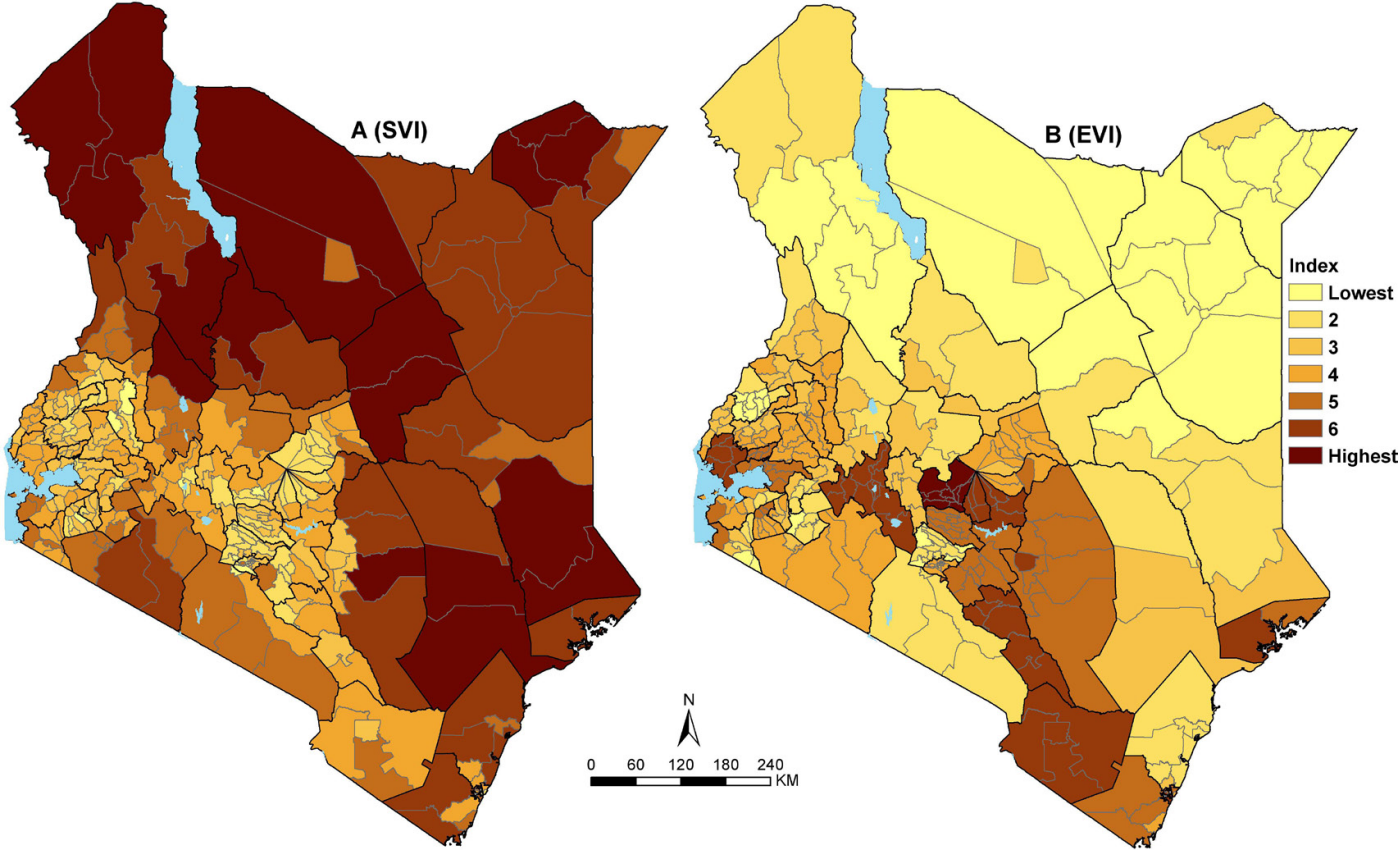
Social Vulnerability Index (SVI) (A) and Epidemiological Vulnerability Index (EVI) (B) across 295 subcounties in Kenya grouped into seven ranks. Ranks 1 and 2 are the least vulnerable subcounties, whereas ranks 6 and 7 are the most vulnerable.

The spatial variation of the SVI was heterogeneous across the 295 subcounties (figure 2A). The least vulnerable subcounties are mainly located in the western and central parts of Kenya, whereas subcounties in northeastern and southern parts are moderately vulnerable. Northwestern and parts of eastern Kenya have the most vulnerable subcounties (figure 2A). Approximately 15% (6.9 million) of Kenya’s total population resides in the 49 subcounties that were classified as highly vulnerable. Almost two-thirds (65%) of Kenya’s population (30.9 million) live in 188 subcounties that are moderately vulnerable, whereas only circa 21% live in the 58 least vulnerable subcounties.

The most vulnerable subcounties in northwestern and partly eastern Kenya with reference to SVI are characterised by poor geographic access to healthcare services, marginalised in terms of access to the nearest urban areas and economically disadvantaged (mainly poor households, poor access to improved water and sanitation and low education attainment) (online supplementary additional file 2 and online supplementary additional file 3). However, despite being the most vulnerable, the region has a low population density and fewer families with single parents relative to other parts of Kenya (online supplementary additional file 2).

10.1136/bmjgh-2020-003014.supp2 Supplementary data

10.1136/bmjgh-2020-003014.supp3 Supplementary data

The least vulnerable subcounties based on SVI mainly in central and western Kenya have a lower proportion of the poor households, improved access to water and handwashing soaps/detergents, higher education attainment, whereas most settlements are near urban areas and within 2 hours of the nearest hospital (online supplementary additional file 3). Despite having on average low vulnerability, both central and western Kenya subcounties have a high population density and a higher proportion of the elderly population. In addition, subcounties in central Kenya and adjacent areas have a slightly higher number of urban population and families with a single parent (online supplementary additional file 2).

Conversely, based on the EVI, 48 subcounties in central, southeast and partly western Kenya where approximately 7.2 million people reside were the most vulnerable (figure 2B). Approximately 13 million (13.2) people residing in 81 subcounties mainly located in the northern and eastern parts were classified as the least vulnerable. The majority of the subcounties in the south of the equator have higher epidemiological vulnerability mainly driven by a high prevalence of hypertension and smoking. High prevalence of obesity and diabetes is only evident in fewer subcounties around central and southeast Kenya, whereas HIV is more prevalent in western Kenya (online supplementary additional file 2 and online supplementary additional file 3). There are exceptions in northeastern, where Mandera county has a higher prevalence for both diabetes and hypertension, whereas Turkana county has a higher prevalence of smoking (online supplementary additional file 2).

**Figure 3:**
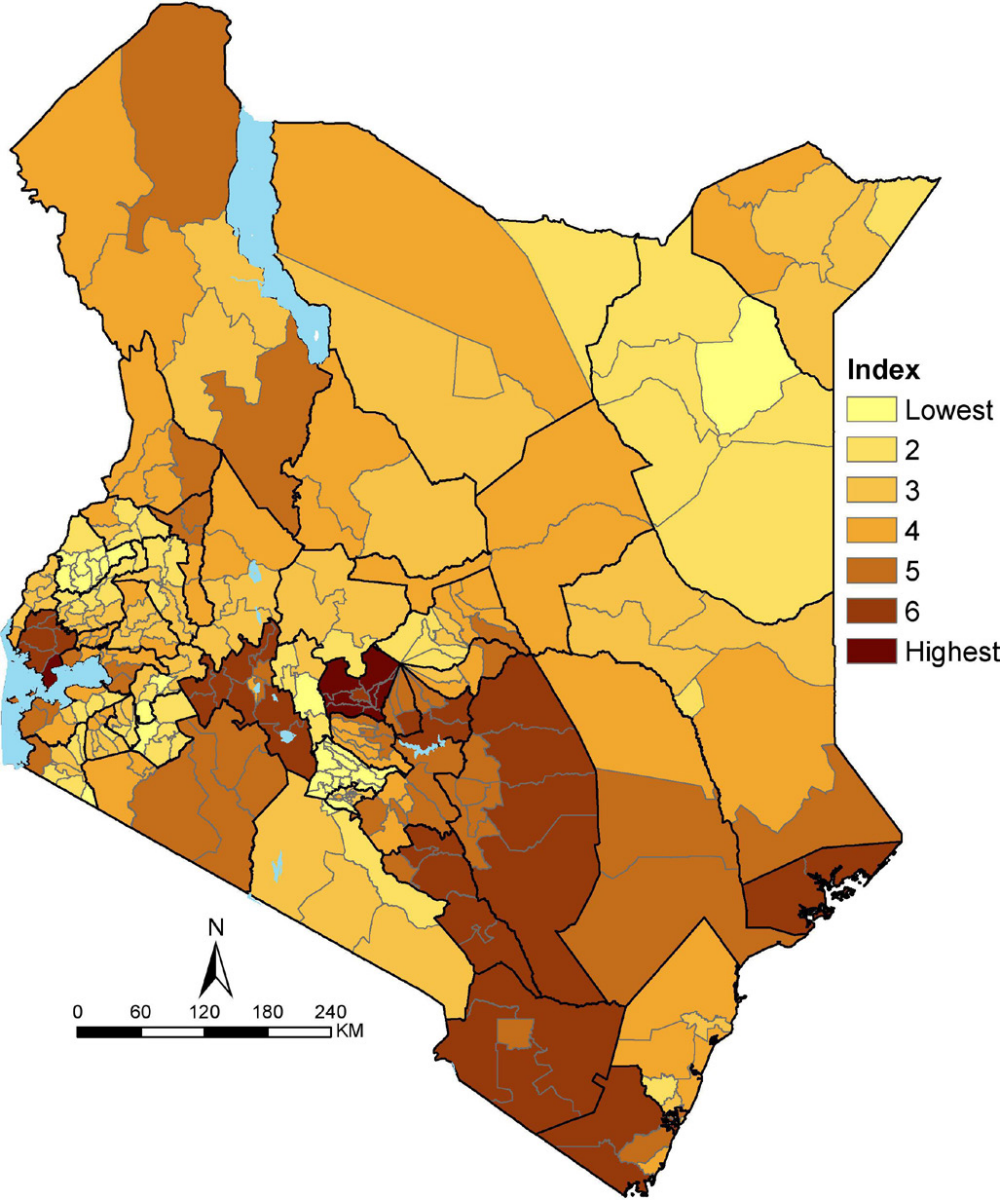
Social Epidemiological Vulnerability Index across 295 subcounties in Kenya grouped into seven ranks. Ranks 1 and 2 are the least vulnerable subcounties, whereas ranks 6 and 7 are the most vulnerable.

The spatial variation of the overlay between SVI and EVI, that is SEVI, is shown in figure 3. The resulting patterns are smoother than EVI and SVI as its average of the two which had fewer areas of the concurrence of the high or low vulnerable subcounties. Consequently, the majority of Kenya’s population (55%) reside in moderately vulnerable subcounties mainly in northwestern and eastern parts of Kenya.

Forty-six subcounties in the central and adjacent areas, southeast and partly western Kenya were the most vulnerable, affecting 15% (7.0 million) of Kenya’s population. More importantly, a few subcounties that are highly vulnerable due to SEVI have a dual burden of SVI and EVI. The notable co-occurrence of EVI and SVI includes parts of Kitui (central-east), Elgeyo Marakwet (partly western) and Narok (southwest). These areas have a high prevalence of smoking, hypertension and stunting, higher proportion of elderly population, low access to improved water and sanitation, and smaller proportion of people within 2 hours of the nearest hospital.

Approximately 30% of the population reside in subcounties classified as the least vulnerable based on SEVI. These subcounties are not localised in one geographic but scattered across Kenya mainly in western (eg, Bungoma County) and a few in northeastern (eg, Wajir County) and central (eg, Kiambu County) Kenya. The localised areas of low vulnerability have different variable factors contributing to the least vulnerable score. For example, in Kiambu, central Kenya, all 24 indicators have low scores (meaning least vulnerable) except five, namely high population density, high urban population, elderly population, shared toilets and high prevalence of smoking whose effect is masked in the combined index. In contrast, Wajir County in northeastern, only crowded and poor households, poor spatial access to hospitals, water and sanitation had higher scores (meaning more vulnerable) whose effect was neutralised by low scores from the rest of the indicators.

## Discussion

Emerging and re-emerging diseases with pandemic potential continue to challenge countries and health systems, causing enormous human and economic losses.^1 3^ Health security calls for the need for all countries to invest in improving their global health preparedness in the phase of emerging epidemics including stronger health infrastructure as the best defence against disease outbreaks and other health threats.^2 73^ Not surprisingly, most countries are struggling to mitigate the impact of the current COVID-19 pandemic with varied levels of success with increasing fears or re-emergence in those places that have managed to contain the pandemic.^74^ It remains uncertain how Africa is going to come through this pandemic. Compared with the rest of the world, the virus has arrived later, providing an opportunity to learn from other contexts that could help guide Africa’s fight. Social distancing and basic hygiene measures are proving to be the most effective tools to slow down the rate of transmission. Yet, in many contexts throughout Africa, social distancing and frequent handwashing are privileges that not everyone has access to.^7 75^

In Kenya, the expectation is that nearly all communities will be affected by COVID-19 to yet undetermined degrees; however, the impact of the pandemic will not be the same in each locality. Meanwhile, Kenya does not have a pre-existing granular vulnerability index such as the CDC’s US SVI.^27^ Such an index would have been used to assess vulnerability or identify vulnerable populations that can be used to inform decisions on the disbursement of social support measures or identify those areas that require improved health services. In this analysis, three indices have been developed to identify which communities need the most support as COVID-19 spreads in the country. Mapped to the subcounty, the vulnerability indices provide information that is useful for emergency response planning and mitigation at a relatively granular level and can help support response planning for the current epidemic.

Once introduced, outbreaks spread faster in vulnerable communities than in less vulnerable areas. Some communities in Kenya were identified to be more vulnerable than others and would exhibit compromised ability to manage the spread and limit the economic and social impact of the outbreak. The SVI identified subcounties in the northern and southeast parts of Kenya as the most vulnerable, whereas the majority of the subcounties in central and western Kenya were observed to be less vulnerable (figure 2A). The 49 most vulnerable subcounties account for 6.9 million (15%) of Kenya’s total population will require greater focus and prioritisation in terms of response.

There is a divergence between subcounties that had high SVI and those defined as epidemiologically most vulnerable (figure 2). Most of the subcounties in western, central and parts of southeast were observed to have high EVI hence when applied to COVID-19, these areas are likely to have subpopulations at higher risk of developing severe disease and increased mortality rates.^18 32–35^ Although the Kenyan population has hypertension, obesity and diabetes, the prevalence is generally not high compared with other settings where severe disease and increased mortality have been observed. Conversely, these regions appear to be less vulnerable with respect to social vulnerability (figure 2A).

Though sparsely populated, northern and southeastern parts of Kenya were less epidemiologically vulnerable and are therefore generally less vulnerable to severe diseases but more susceptible to infections and spread when considering their socioeconomic context. They have poor access to hospitals and urban areas, high number of poor households, constrained access to water and sanitation, and low education attainment. These metrics allow for the identification of geographic areas that are most likely to harbour large numbers of undocumented COVID-19 cases due to lack of access to care (online supplementary additional file 2, online supplementary additional file 3), which in turn can inform the geographic targeting of testing and surveillance efforts and for the deployment of temporary hospitals based on projected need.

The government of Kenya has put in place several measures to curb the spread of COVID-19. Some of these measures lead to reduced social interaction hence reduced production and demand across all the sectors that are costly to the economy and will have negative impacts on the livelihoods of people. The national government, the county governments and other stakeholders are implementing programmes to cushion against adverse socioeconomic impacts. These include reduction of taxes, provision of masks and hand sanitisers, distribution of food, water and other commodities. Further, the government will inject Kenya shillings 53.7 billion into the economy to stimulate growth and cushion families and companies during COVID-19 pandemic in eight thematic areas including infrastructure, education, small and medium enterprises, health, agriculture, tourism, environment and manufacturing.^76^ This analysis identifies areas that should be prioritised for different interventions when programmes to ease vulnerability and mitigate the effects of COVID-19 are being rolled out across the country. This is important especially in areas that are highly vulnerable and are yet to experience an escalation of cases. These indices have the potential to pave the way for data-driven informed planning to tackle vulnerability.

The epidemic is shaped by many factors, testing capacity and social distancing, as well as population density, age structure, wealth and other social behavioural factors. In Kenya, the spread of the virus is uneven with most of the cases identified in the capital city, larger towns and a few border towns. By overlaying the number of confirmed cases of COVID-19 onto the vulnerability indices, we can begin to explore the spread of the virus in communities with different levels of vulnerability. We have preliminarily explored how vulnerability relates to the numbers of confirmed cases of COVID-19 using case data at the county level made available by May 14. Identified hotspots (Mvita subcounty, Dagoretti north and Kamukunji subcounties) are in highly vulnerable areas when considering their population characteristics that include being largely urban, high population density with a high proportion of people living within informal settlements. Further, they have most people within informal employment and shared sanitation facilities. This is somewhat confounded by the fact that we are unable to separate imported cases which comprise a substantial percentage of the total case count.^77^

Countries are starting to ease lockdown measures to limit the negative impact on the economy.^74 78^ These decisions need to be informed by the trends in new cases, the potential risk of resurgence and the strength of public health systems including the capacity to detect new cases. The vulnerability index constitutes a measure through which to better appreciate factors that enable communities to remain resilient, inform on their ability to carry out personal protective measures, practice both hand hygiene and hygiene in the household and the possibility of social distancing in a different context. Importantly, these indices have identified indicators that shed light on factors that would drive the continued spread of disease and inform prediction on the burden of severe disease and mortality due to COVID-19 in Kenya. Importantly, the indices developed are versatile and can be repurposed for use in varied emergency response situations.

The indices showed widespread inequities across subcounties of Kenya. Although the interim measures will help ease the pressure and reduce vulnerability, there is a need to reduce inequities in the longer term, beyond the current COVID-19 pandemic in preparation for future epidemics that are inevitable.^79^ Africa must invest heavily in relevant data systems and preparedness by increased government investments. Programmes to ensure access to improved water and sanitation, targeted social programmes such as raising awareness for proper hygiene, improvement of housing facilities in the informal settlements and IDP camps are needed. Strengthening health systems remains at the core of reducing health inequities.^79^ The system should also be redesigned to deal with surge capacity by absorbing the increase in the demand for healthcare services due to epidemics and pandemics.^31^

The prevalence and recurrence of epidemics and disasters in Africa should be the impetus for greater investment in preparedness. Disasters such as COVID-19 pandemic,^80 81^ Ebola epidemic,^4^ flooding in east and southern Africa,^82^ ongoing floods and recurrent malaria epidemics in Kenya^83^ have become common. Investments on measures such as early warning systems should be put into place to detect, respond and effectively contain these threats. Strategic actions that were recommended against influenza could potentially inform better preparation in case of a viral disease: capability to develop pandemic strain vaccines, stockpiles of broad-spectrum antiviral drugs, surge capacity for rapid vaccine production and developing models that could inform effective means of delivering therapies during an outbreak.^84^

The indicators used in this analysis to derive vulnerability indices were based on the a priori understandings of who is a risk and vulnerable from information available so far, however, COVID-19 is evolving and more insights will become increasingly available and the indices can be adapted. Further, the classification of indicators into sub domains should be adapted to context and data availability. For example, Wilkinson *et al* proposes categorising the indicators into epidemiological vulnerability (based on underlying health conditions), transmission vulnerability (eg, social mixing and hygiene infrastructure), health system vulnerability (eg, hospitals beds and health workers and so on) and vulnerability to control measures.^85^ In addition, there is a paucity of information on outcomes related to epidemiological factors in African countries, yet this will improve over time as more data become available. As we learn more about COVID-19 disease outcomes in these contexts, it will be important to adjust the created index to incorporate any observed differences.

### Limitations

There were data-related limitations when computing the vulnerability indices. Several variables such as access to mobile phones, access to insurance cover and mobility between counties could not be accessed during the analysis. Further, due to the lack of granular data despite using SAE techniques, obesity, diabetes and hypertension could only be resolved at the county level. Therefore, we assumed that the estimates within subcounty were equal for these three variables. Some of the datasets are not updated to 2020 and their trends are likely to have changed between 2014 and 2020. Furthermore, the analysis was conducted at the subcounty level; it is likely that some variation and heterogeneity were masked in the relatively bigger polygons.

An equal weighting scheme was implemented for all the determinants as routinely applied.^67–70^ Equal weighting schemes have shown to be equally robust;^56 58 60 61 65 71^ however, the weighting scheme can be revised as more individual-level COVID-19 data with attributes become available in Kenya and other countries across Africa. This will allow a better definition of how the risks associated with different variables vary across different populations and settings.^18^

The preliminary overlay between indices and the cases may have been limited. Cases were allocated in counties where they were recorded; however, these might not have been the residential counties for the last several years. Some might have been living outside Kenya and other counties. Furthermore, some cases were imported from other countries.^77^ In contrast, the data layers used refer to the specific counties for the period between 2014 and 2020.

## Conclusions

Fighting the COVID-19 pandemic calls for precision public health reflecting our improved understanding of who is most vulnerable and what makes them more vulnerable, where the disease is spreading or likely to spread fastest, and where current interventions may not work as intended. COVID-19 is spreading at different rates across Kenya, most probably working its way through all 47 counties. The indices estimated presents tools that can be used by the Kenyan government and stakeholders to a better plan by prioritising subcounties that are moderate to highly vulnerable. The heterogeneous nature of the indices further underpins the need to start intervening by prioritising the subcounties with the greatest needs.
