# Supplementary material for: A vulnerability index for COVID-19: spatial analysis at the subnational level in Kenya
Source: BMJ Glob Health. 2020 Aug 23;5(8):e003014. doi: 10.1136/bmjgh-2020-003014 (PMC7447114; doi:10.1136/bmjgh-2020-003014)
Supplement: Supplementary data [file bmjgh-2020-003014supp001.pdf]

Additional file 1: The county and sub counties of Kenya

Figure1: The map of Kenya showing 47 counties (colored) and 295 sub-counties (numbered). The extents of major lakes and the Indian Ocean are shown in light blue. The names of the counties and sub-counties corresponding to the shown numbers below the maps.

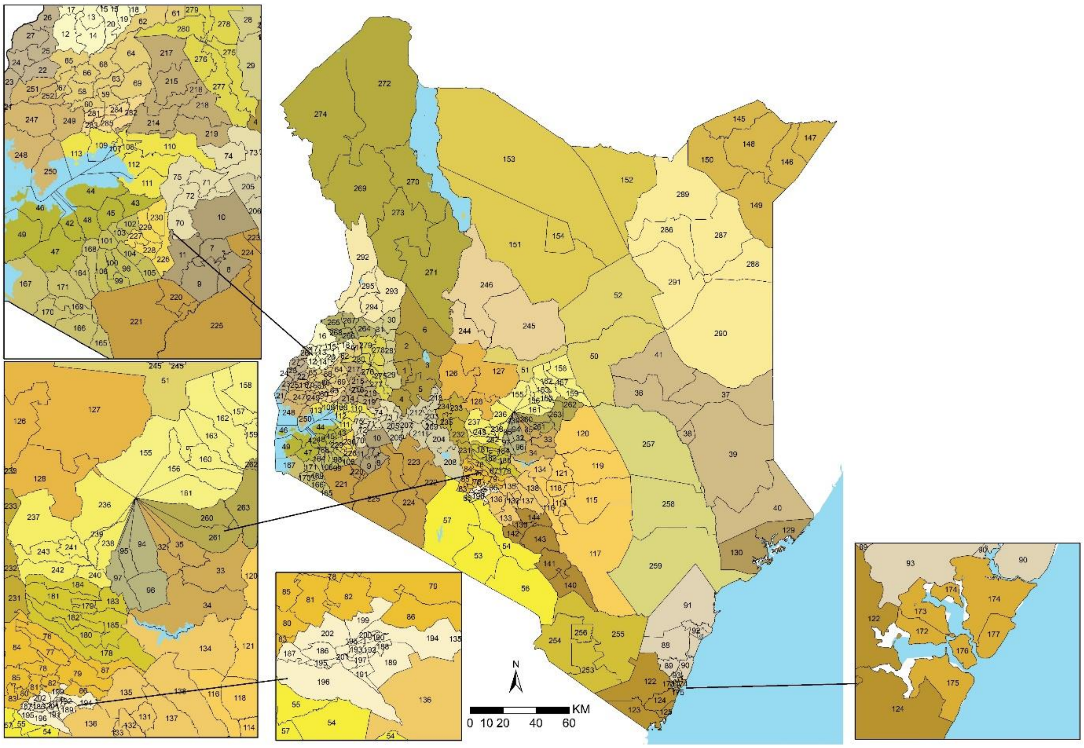

List of Counties (bold) and their respective sub county (numbered) as presented in Figure 1

1. **Baringo county**: Baringo Central [1], Baringo North [2], Baringo South [3], Eldama Ravine [4], Mogotio [5], Tiaty [6]

2. **Bomet county**: Bomet Central [7], Bomet East [8], Chepalungu [9], Konoin [10], Sotik [11]

3. **Bungoma county**: Bumula [12], Kabuchai [13], Kanduyi [14], Kimilili [15], Mt Elgon [16], Sirisia [17], Tongaren [18], Webuye East [19], Webuye West [20]

4. **Busia county**: Budalangi [21], Butula [22], Funyula [23], Matayos [24], Nambale [25], Teso North [26], Teso South [27]

5. **Elgeyo Marakwet county**: Keiyo North [28], Keiyo South [29], Marakwet East [30], Marakwet West [31]

6. **Embu county**: Manyatta [32], Mbeere North [33], Mbeere South [34], Runyenjes [35]

7. **Garissa**: Balambala [36], Dadaab [37], Dujis [38], Fafi [39], Ijara [40], Lagdera [41]

8. **Homa Bay county:** Homa Bay [42], Kabondo Kasipul [43], Karachuonyo [44], Kasipul [45], Mbita [46], Ndhiwa [47], Rangwe [48], Suba [49],

9. **Isiolo county**: Garbatulla [50], Isiolo [51], Merti [52]

10. **Kajiado county**: Kajiado Central [53], Kajiado East [54], Kajiado North [55], Kajiado South [56], Kajiado West [57]

11. **Kakamega county**: Butere [58], Ikolomani [59], Khwisero [60], Likuyani [61], Lugari [62], Lurambi [63], Malava [64], Matungu [65], Mumias East [66], Mumias West [67], Navakholo [68], Shinyalu [69]

12. **Kericho county**: Buret [70], Kericho East [71], Kericho West [72], Kipkelion East [73], Kipkelion West [74], Sigowet [75]

13. **Kiambu county**: Gatundu North [76], Gatundu South [77], Githunguri [78], Juja [79], Kabete [80], Kiambaa [81], Kiambu [82], Kikuyu [83], Lari [84], Limuru [85], Ruiru [86], Thika Town [87]

14. **Kilifi county**: Ganze [88], Kaloleni [89], Kilifi [90], Magarini [91], Malindi [92], Rabai [93],

15. **Kirinyaga county**: Gichugu [94], Kirinyaga Central [95], Mwea [96], Ndia [97]

16. **Kisii county**: Bobasi [98], Bomachoge Borabu [99], Bomachoge Chache [100], Bonchari [101], Kitutu Chache North [102], Kitutu Chache South [103], Nyaribari Chache [104], Nyaribari Masaba [105], South Mugirango [106]
17. **Kisumu county**: Kisumu Central [107], Kisumu East [108], Kisumu West [109], Muhoroni [110], Nyakach [111], Nyando [112], Seme [113]

18. **Kitui county**: Kitui Central [114], Kitui East [115], Kitui Rural [116], Kitui South [117], Kitui West [118], Mwingi East [119], Mwingi North [120], Mwingi West [121]

19. **Kwale county**: Kinango [122], Lunga Lunga [123], Matuga [124], Msambweni [125]

20. **Laikipia county**: Laikipia East [126], Laikipia North [127], Laikipia West [128]

21. **Lamu county:** Lamu East [129], Lamu West [130]

22. **Machakos county**: Kangundo [131], Kathiani [132], Machakos Town [133], Masinga [134], Matungulu [135], Mavoko [136], Mwala [137], Yatta [138]

23. **Makueni county**: Kaiti [139], Kibwezi East [140], Kibwezi West [141], Kilome [142], Makueni [143], Mbooni [144]

24. **Mandera county**: Banissa [145], Lafey [146], Mandera East [147], Mandera North [148], Mandera South [149], Mandera West [150]

25. **Marsabit county**: Laisamis [151], Moyale [152], North Horr [153], Saku [154],

26. **Meru county**: Buuri [155], Central Imenti [156], Igembe Central [157], Igembe North [158], Igembe South [159], North Imenti [160], South Imenti [161], Tigania East [162], Tigania West [163]

27. **Migori county**: Awendo [164], Kuria East [165], Kuria West [166], Nyatike [167], Rongo [168], Suna East [169], Suna West [170], Uriri [171]

28. **Mombasa county**: Changamwe [172], Jomvu [173], Kisauni [174], Likoni [175], Mvita [176], Nyali [177]

29. **Murang'a county**: Gatanga [178], Kahuro [179], Kandara [180], Kangema [181], Kigumo [182], Kiharu [183], Mathioya [184], Murang'a South [185]

30. **Nairobi county**: Dagoretti North [186], Dagoretti South [187], Embakasi Central [188], Embakasi East [189], Embakasi North [190], Embakasi South [191], Embakasi West [192], Kamukunji [193], Kasarani [194], Kibra [195], Langata [196], Makadara [197], Mathare [198], Roysambu [199], Ruaraka [200], Starehe [201], Westlands [202]

31. **Nakuru county**: Bahati [203], Gilgil [204], Kuresoi North [205], Kuresoi South [206], Molo [207], Naivasha [208], Nakuru Town East [209], Nakuru Town West [210], Njoro [211], Rongai [212], Subukia [213]

32. **Nandi county**: Aldai [214], Chesumei [215], Emgwen [216], Mosop [217], Nandi Hills [218], Tinderet [219]
33. **Narok county**: Emurua Dikirr [220], Kilgoris [221], Narok East [222], Narok North [223], Narok South [224], Narok West [225],

34. **Nyamira county**: Borabu [226], Manga [227], Masaba North [228], Nyamira [229], Nyamira North [230]

35. **Nyandarua county**: Kinangop [231], Kipipiri [232], Ndaragwa [233], Ol Jorok [234], Ol Kalou [235]

36. **Nyeri county**: Kieni East [236], Kieni West [237], Mathira East [238], Mathira West [239], Mukurweini [240], Nyeri Town [241], Othaya [242], Tetu [243]

37. **Samburu county**: Samburu Central [244], Samburu East [245], Samburu North [246],

38. **Siaya county:** Alego Usonga [247], Bondo [248], Gem [249], Rarieda [250], Ugenya [251], Ugunja [252]

39. **Taita Taveta county**: Mwatate [253], Taveta [254], Voi [255], Wundanyi [256]

40. **Tana River county**: Bura [257], Galole [258], Garsen [259]

41. **Tharaka Nithi county**: Maara [260], Meru South [261], Tharaka North [262], Tharaka South [263]

42. **Trans Nzoia county**: Cherangany [264], Endebess [265], Kiminini [266], Kwanza [267], Saboti [268],

43. **Turkana county**: Loima [269], Turkana Central [270], Turkana East [271], Turkana North [272], Turkana South [273], Turkana West [274],

44. **Uasin Gishu county**: Ainabkoi [275], Kapseret [276], Kesses [277], Moiben [278], Soy [279], Turbo [280]

45. **Vihiga county**: Emuhaya [281], Hamisi [282], Luanda [283], Sabatia [284], Vihiga [285],

46. **Wajir county**: Eldas [286], Tarbaj [287], Wajir East [288], Wajir North [289], Wajir South [290], Wajir West [291]

47. **West Pokot county**: North Pokot [292], Pokot Central [293], Pokot South [294], West Pokot [295].
