# Supplementary material for: A vulnerability index for COVID-19: spatial analysis at the subnational level in Kenya
Source: BMJ Glob Health. 2020 Aug 23;5(8):e003014. doi: 10.1136/bmjgh-2020-003014 (PMC7447114; doi:10.1136/bmjgh-2020-003014)

BMJ Publishing Group Limited (BMJ) disclaims all liability and responsibility arising from any reliance placed on this supplemental material which has been supplied by the author(s)

Socioeconomic inequality- scaled indicators

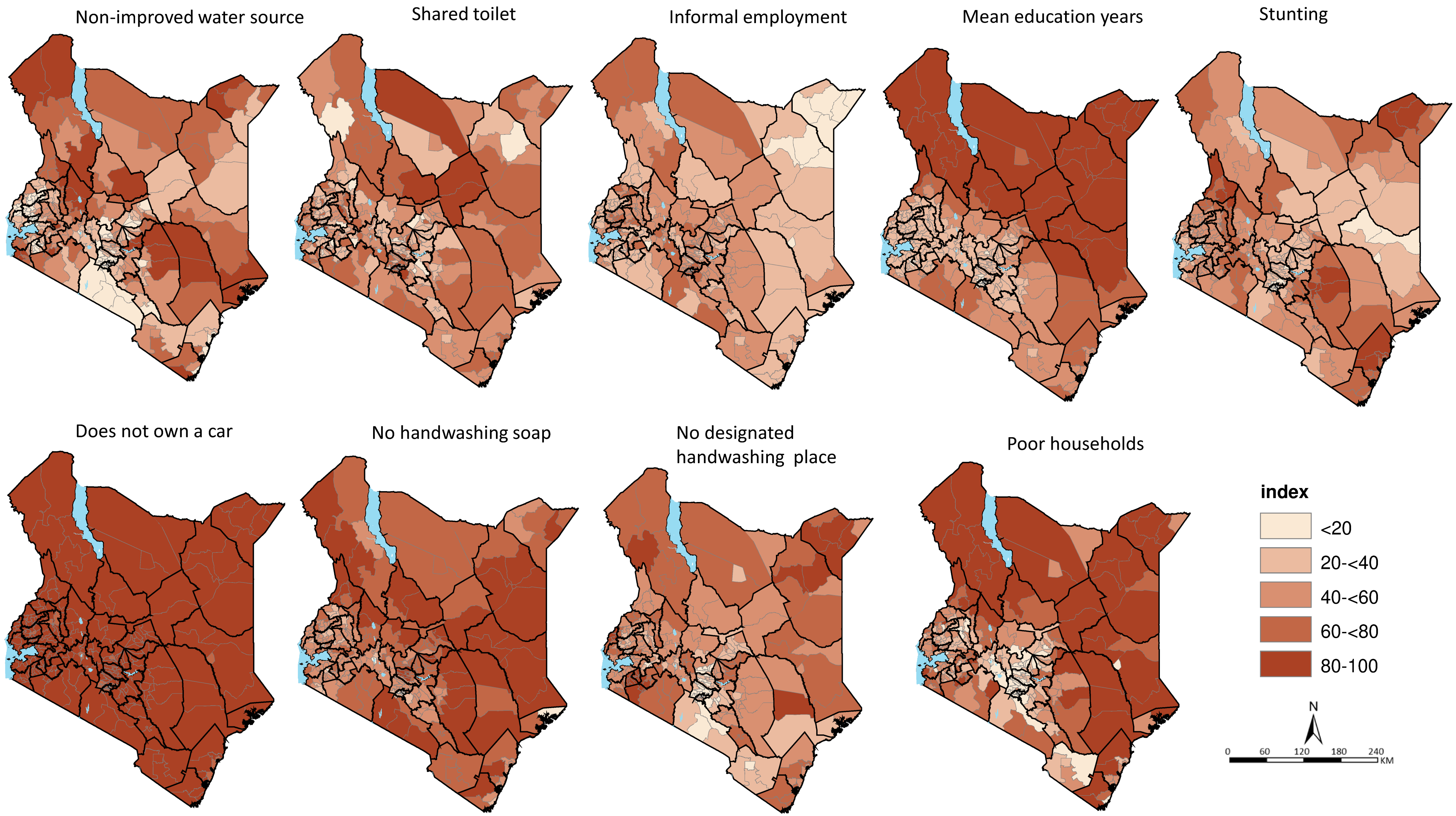

BMJ Publishing Group Limited (BMJ) disclaims all liability and responsibility arising from any reliance placed on this supplemental material which has been supplied by the author(s)

Access to services – scaled indicators

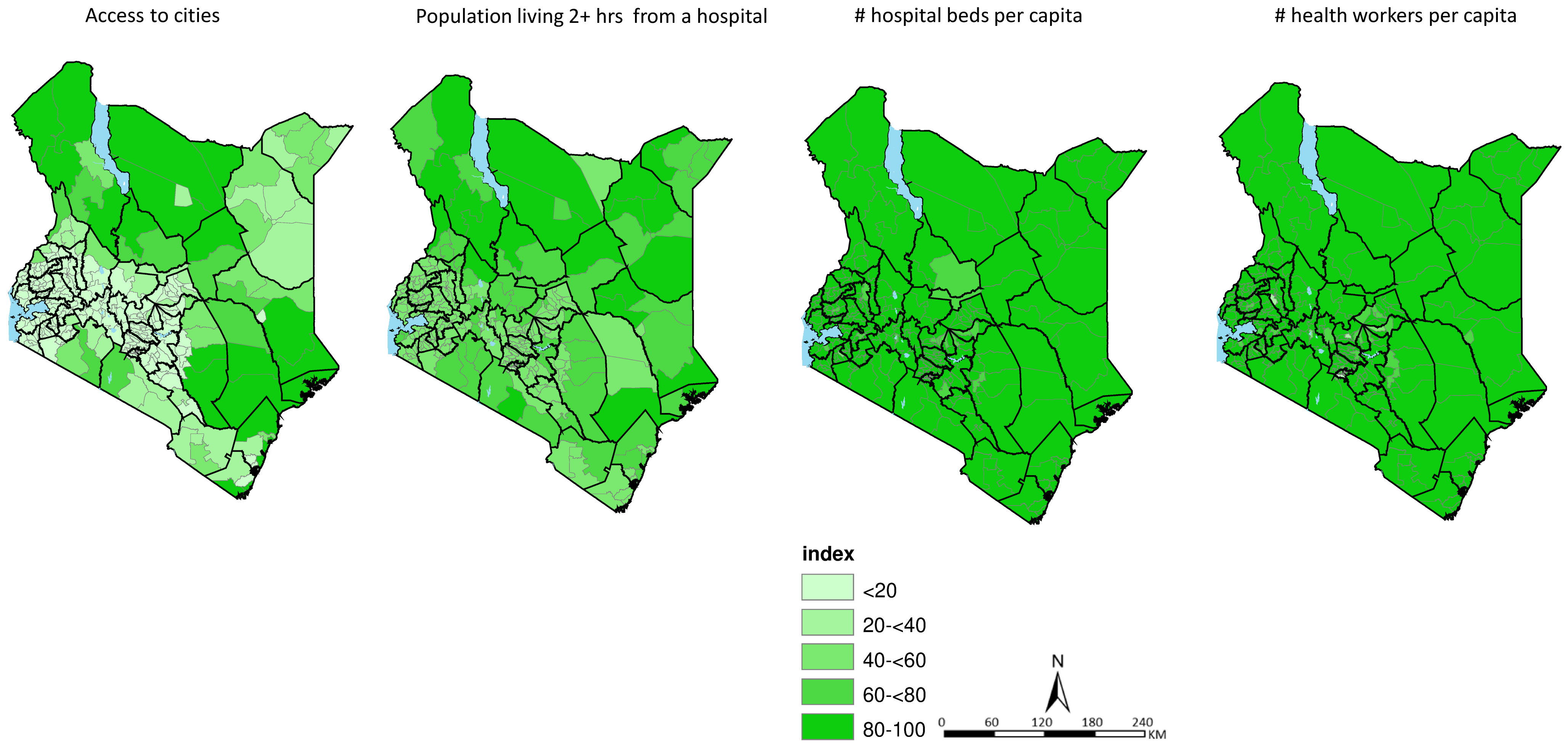

Population characteristics –Scaled indicators

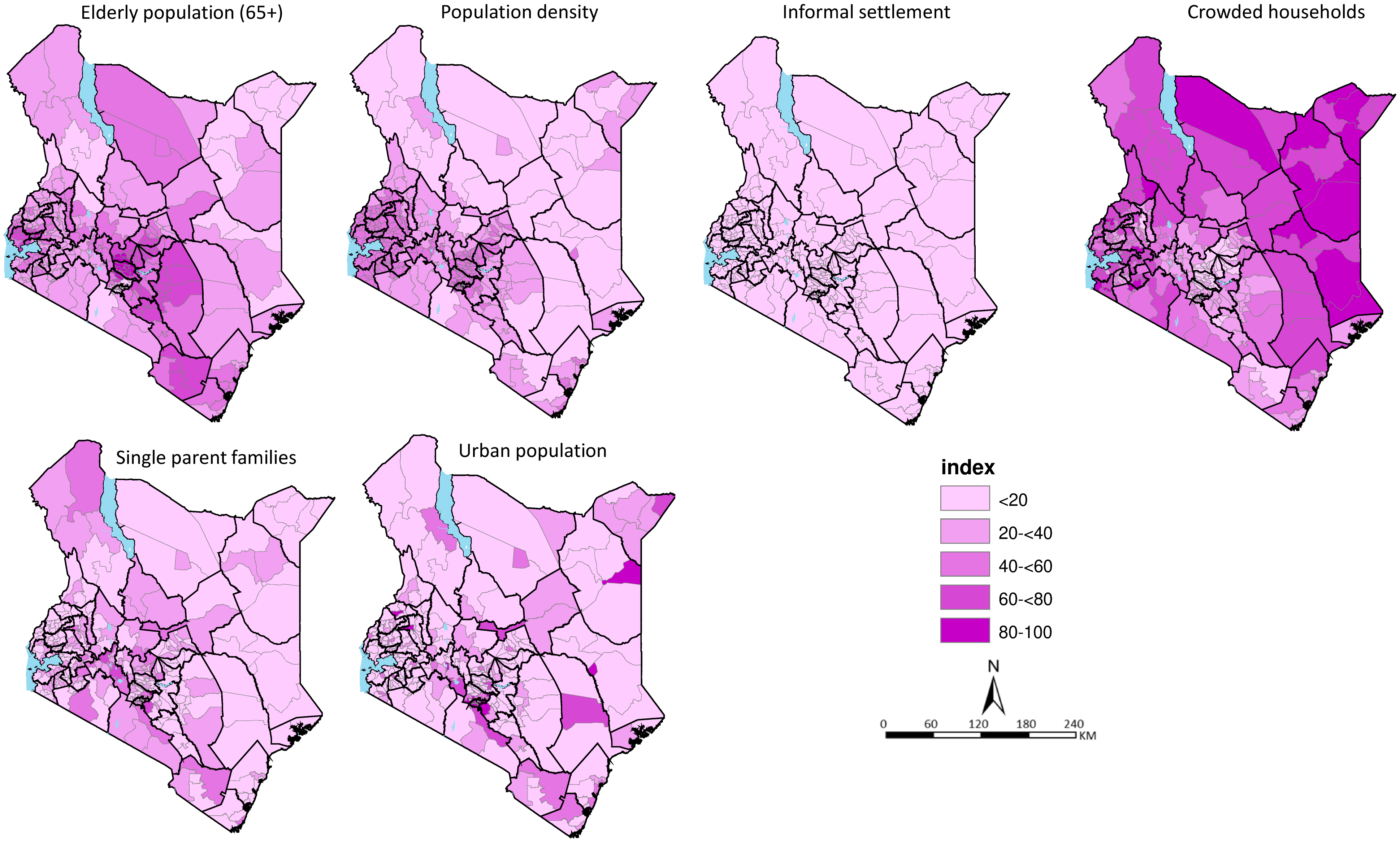

BMJ Publishing Group Limited (BMJ) disclaims all liability and responsibility arising from any reliance placed on this supplemental material which has been supplied by the author(s)

Epidemiological factors –Scaled indicators

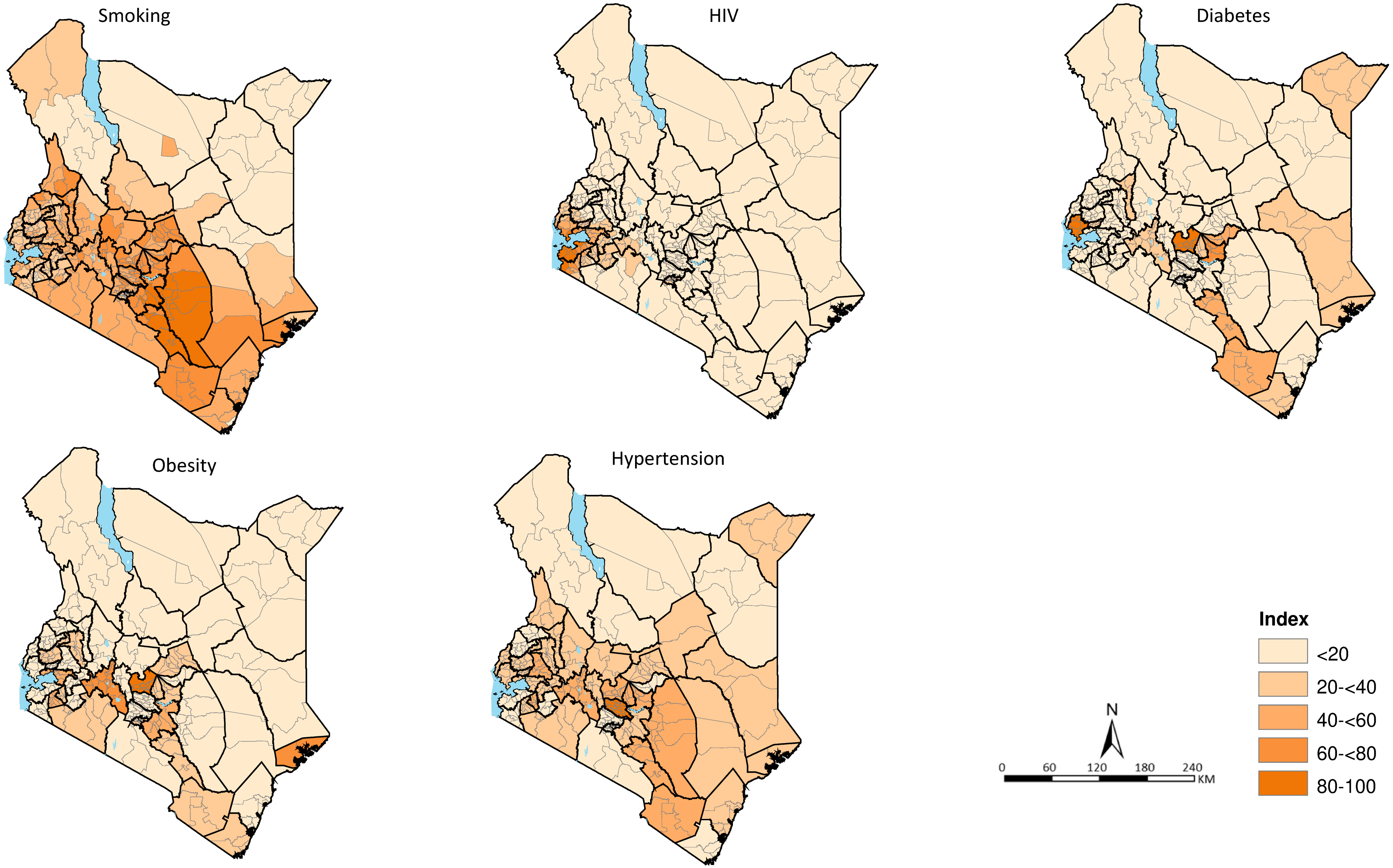

Supplement: Supplementary data [file bmjgh-2020-003014supp002.pdf]
