# Supplementary material for: A vulnerability index for COVID-19: spatial analysis at the subnational level in Kenya
Source: BMJ Glob Health. 2020 Aug 23;5(8):e003014. doi: 10.1136/bmjgh-2020-003014 (PMC7447114; doi:10.1136/bmjgh-2020-003014)

Three sub domain indices used to define the social vulnerability index across the 295 sub counties of Kenya; Socioeconomic deprivation index (A), population characteristics index (B), access to services index (C). Each index is grouped into seven ranks. Rank 1 and 2 are the least vulnerable sub counties while rank 6 and 7 are the most vulnerable sub counties.

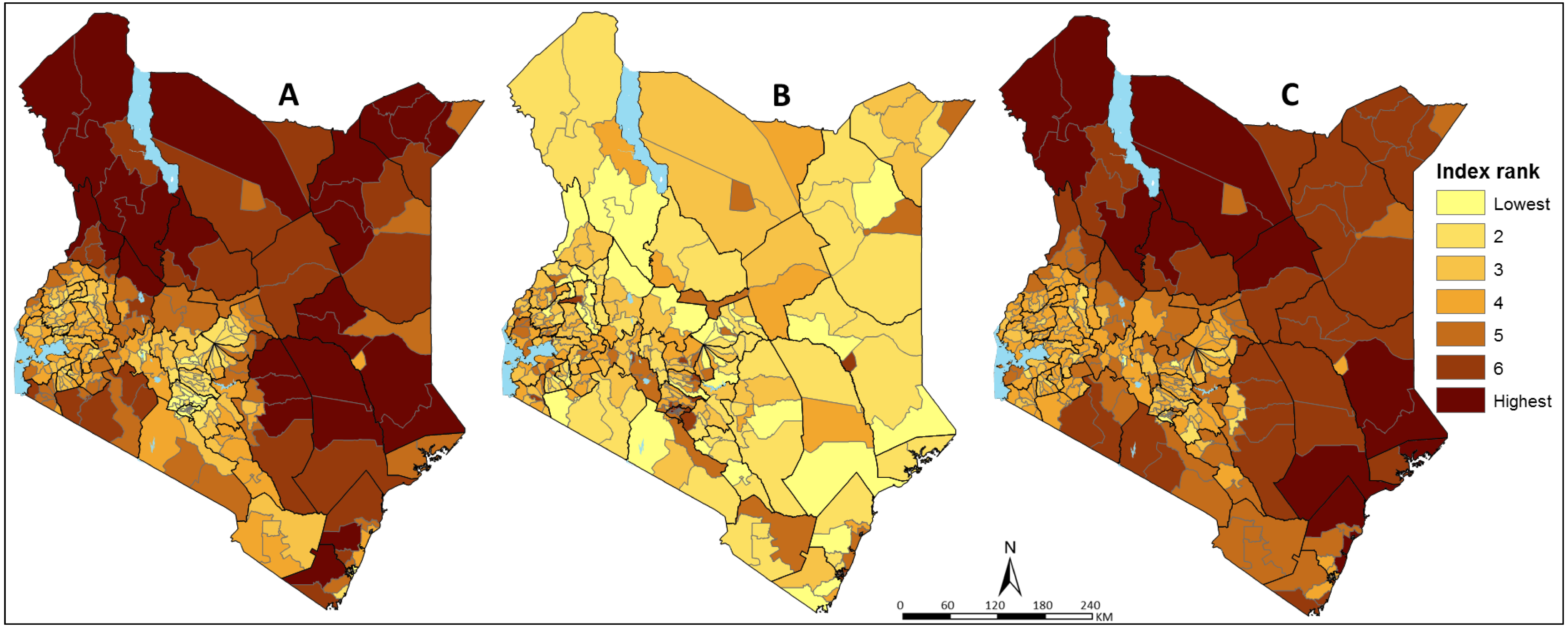

Supplement: Supplementary data [file bmjgh-2020-003014supp003.pdf]
